# TNFSF15 facilitates the differentiation of CD11b^+^ myeloid cells into vascular pericytes in tumors

**DOI:** 10.20892/j.issn.2095-3941.2023.0245

**Published:** 2023-11-02

**Authors:** Xiangxiang Gu, Yipan Zhu, Cancan Zhao, Yixin Cao, Jingying Wang, Qiangzhe Zhang, Luyuan Li

**Affiliations:** State Key Laboratory of Medicinal Chemical Biology and College of Pharmacy, Nankai University, and Haihe Laboratory of Cell Ecosystem, Tianjin 300350, China

**Keywords:** TNFSF15, myeloid cell, neovascularization, CD11b^+^ cell, pericyte

## Abstract

**Objective:**

Immature vasculature lacking pericyte coverage substantially contributes to tumor growth, drug resistance, and cancer cell dissemination. We previously demonstrated that tumor necrosis factor superfamily 15 (TNFSF15) is a cytokine with important roles in modulating hematopoiesis and vascular homeostasis. The main purpose of this study was to explore whether TNFSF15 might promote freshly isolated myeloid cells to differentiate into CD11b^+^ cells and further into pericytes.

**Methods:**

A model of Lewis lung cancer was established in mice with red fluorescent bone marrow. After TNFSF15 treatment, CD11b^+^ myeloid cells and vascular pericytes in the tumors, and the co-localization of pericytes and vascular endothelial cells, were assessed. Additionally, CD11b^+^ cells were isolated from wild-type mice and treated with TNFSF15 to determine the effects on the differentiation of these cells.

**Results:**

We observed elevated percentages of bone marrow-derived CD11b^+^ myeloid cells and vascular pericytes in TNFSF15-treated tumors, and the latter cells co-localized with vascular endothelial cells. TNFSF15 protected against CD11b^+^ cell apoptosis and facilitated the differentiation of these cells into pericytes by down-regulating Wnt3a-VEGFR1 and up-regulating CD49e-FN signaling pathways.

**Conclusions:**

TNFSF15 facilitates the production of CD11b^+^ cells in the bone marrow and promotes the differentiation of these cells into pericytes, which may stabilize the tumor neovasculature.

## Introduction

The tumor neovasculature is characterized by an abnormal vascular wall structure that lacks pericyte support^1,2^ and consequently has poor ability to sustain normal circulation^3–5^, thus resulting in persistent hypoxic conditions and inflammation in tumors^6,7^. Restoration of the tumor vasculature with improved pericyte coverage has therefore been a focus of research in the development of tumor therapeutic approaches^8–11^. Analyses of the sources of pericytes in tumors have indicated that myeloid-derived CD11b^+^ cells that infiltrate into tumors participate in tumor neovascularization^12,13^. Instead of differentiating into endothelial cells, tumor-infiltrated CD11b^+^ cells are recruited to the blood vessels, where they act as pericytes in tumor neovascularization^14,15^. However, the mechanisms driving this process remain unclear.

Tumor necrosis factor superfamily 15 (TNFSF15, also known as VEGI^16^ or TL1A^17^) is produced largely by vascular endothelial cells in established blood vessels in normal tissues but is only marginally expressed in the tumor vasculature in a variety of cancers^18–20^, as well as in wound tissues^21^. TNFSF15 appears to function primarily in the maintenance of vascular homeostasis^22–26^. We have shown that TNFSF15 inhibits bone marrow-derived hematopoietic stem cell differentiation into endothelial progenitors and endothelial cells, thus inhibiting endothelial progenitor cell-driven vasculogenesis^27–29^. Additionally, TNFSF15 has been found to facilitate lymphatic vessel growth in animal models^30^ and to promote naive or M2 macrophage polarization into M1 macrophages^31^. These findings indicate that TNFSF15 plays an important role in modulating myeloid cell differentiation.

In this study, we show that TNFSF15 stimulates the production of CD11b^+^ myeloid cells in the bone marrow, and facilitates bone marrow-derived CD11b^+^ cell differentiation into pericytes in both cell cultures and in animal models. Furthermore, treatment of tumor-bearing mice with recombinant TNFSF15 enhances the association of myeloid cell-derived pericytes with endothelial cells in tumor vessels, thereby normalizing the wall structure of the new blood vessels. These findings provide new insights into the role of myeloid cells in neovascularization.

## Materials and methods

### Cells and reagents

Lewis lung carcinoma (LLC) cells were purchased from the American Type Culture Collection (Manassas, VA). Cells were cultured in Dulbecco’s modified Eagle’s medium (Lonza, Walkersville, MD) supplemented with 10% fetal bovine serum (Gemini Bio-Products, West Sacramento, CA). TNFSF15 and 4–3H were prepared in our laboratory^32,33^. One unit of TNFSF15 activity was defined as the IC50 of the preparation on bovine aortic endothelial cell proliferation, i.e., the concentration of TNFSF15 required for half-maximum inhibition of cell growth in culture.

### Experimental animals

C57BL/6 mice (6–8 weeks old) were purchased from Vital River Laboratory Animal Center (Beijing, China), kept under specific-pathogen-free conditions, and given free access to standard food and water. The SPC-TNFSF15 transgenic mouse strain (C57BL/6 background) was established by the laboratory of L.-Y. L.^30^. In this study, 8-week-old female SPC-TNFSF15 transgenic mice and their littermates were used. All procedures involving experimental animals were performed in accordance with protocols approved by Nankai University Care and Use Committee (approval number 2023-SYDWLL-000456).

### Acquisition of bone marrow cells

C57BL/6 mice were sacrificed by CO_2_ asphyxiation and soaked in 75% alcohol for 3–5 min. The lower limb bones of the mice were aseptically extracted on a sterilized laboratory bench. The abdominal skin between the hips of the mice was grasped with ophthalmic forceps, the skin was carefully cut with ophthalmic scissors, and the skin of the lower limbs was separated, cut at the ankle inferiorly and the hip superiorly. The 2 lower limbs of the mice were freed. Muscle and connective tissue were carefully dissected, the femur and tibia were dissected, and cartilage was cut off at both ends to expose the red marrow cavity. PBS was aspirated with a sterile 1 mL syringe and gently inserted into the bone marrow cavity, and the bone marrow cavity was repeatedly flushed to obtain bone marrow cells.

### CD11b^+^ cell isolation

CD11b^+^ cells were collected and isolated from mouse bone marrow through flow cytometry. CD11b^+^ cells were sorted with an EasySepTM Mouse CD11b Positive Selection Kit II according to the manufacturer’s instructions (18770, STEMCELL). Cell isolation, culturing, and characterization studies were performed as previously described.

### Red fluorescent TdTomato bone marrow transplantation in LLC tumor-bearing models

Female C57BL/6J mice of SPF grade were exposed to a 9.0 Gry radiation dose for 10 min, and bone marrow transplantation was performed 6 h thereafter. The bone marrow cells of 8-week-old tdTomato male mice were harvested aseptically and injected into the tail veins of the mice. The mice that accepted tdTomato^+^ bone marrow transplantation were subcutaneously inoculated with 5 × 10^5^ LLC cells into the right flank, and tumor size (length × width^2^ × π/6) was monitored every other day. The tumor-bearing mice were randomly divided into 2 groups on day 7, then intraperitoneally injected with recombinant TNFSF15 (5 mg/kg) or buffer every other day, for 7 days.

### Isolation of single cells from murine tumors

The LLC tumors were minced and then enzymatically dissociated in Hanks’ balanced salt solution containing 1 mg/mL collagenase IV (Sigma), 0.1 mg/mL hyaluronidase V (Sigma), and 5 μU/mL DNase I (Sigma) at 37°C for 30 min. Red blood cells were solubilized with red cell lysis buffer (Solarbio), and the resulting suspension was filtered through a 70 μm cell strainer again to produce a single cell suspension for flow cytometry analysis.

### Flow cytometry

Tumor samples and bone marrow were collected for FCM analysis with the corresponding antibodies. The single cell samples were stained with the indicated antibodies for 30 min on ice. Antibodies to the following were used: for CD11b^+^ cells, CD11b (101204, BioLegend); for pericytes, PDGFRβ (323606, BioLegend), α-SMA (MAB1420, R&D Systems), Desmin (ab32362, Abcam), NG2 (ab259324, Abcam), Flt-1 (SC-316, Santa Cruz), CD31 (11-0311-81, eBioscience), CD49e (103805, BioLegend), and Ki-67 (2247496, eBioscience). FlowJo software (version 10.7.4) was used for analysis, and gatings were based on appropriate isotype control staining.

### Cell apoptosis assays

Cell apoptosis assays were performed according to the manufacturer’s recommendations. CD11b^+^ cells from mouse bone marrow were cultured in expansion culture medium for 3 days in the presence of TNFSF15 at 37°C under 5% CO_2_. The cells were collected and washed with PBS, then resuspended with 1× loading buffer, and stained with Annexin V-FITC and PI for 15 min at room temperature in the dark. The cells were then analyzed by flow cytometry.

### RNA extraction and real-time quantitative PCR

Total RNA was extracted from cells and frozen tissues with an RNeasy Mini Kit (Qiagen, Venlo, Netherlands). A Transcriptor High Fidelity cDNA Synthesis Kit (Roche Diagnostics, Roswell, GA, USA) was used for cDNA synthesis. For RT-PCR, cDNA was amplified with a thermal cycler. RT-PCR was performed by amplification of the target genes and Gapdh mRNA as a reference gene with a Light Cycler 480-II (Roche Diagnostics) instrument. The primers used in these experiments are listed in **Supplementary Table S1**.

### Immunofluorescence staining

For immunofluorescence staining, cells were attached to a coverslip, and 4% paraformaldehyde fixed cells were permeabilized for 5 min with 0.2% Triton X-100 in PBS. After incubation with primary antibodies at 4°C for 12 h, the cells were stained with anti-CD31 (ab7388, Abcam), anti-α-SMA (MAB1420, R&D Systems), anti-PDGFR-β (ab69506, Abcam), or anti-Desmin (ab32362, Abcam). The cells were washed with PBS and subsequently reacted with IgG fluorescent secondary antibodies. Cell nuclei were stained with DAPI. The samples were sealed with antifade mounting medium, and images were taken with a Leica TCS SP8 system.

### Cell viability assays

The viability of CD11b^+^ cells was assessed by staining with Calcein-AM assays according to the manufacturer’s instructions. Cells were observed under an inverted fluorescence microscope (Leica Microsystems, Wetzlar, Germany).

### Immunoblotting

Cells with different treatments were washed twice with PBS, then collected and lysed in western IP buffer. The cell lysates were separated on sodium dodecyl sulfate polyacrylamide gels and transferred to polyvinylidene difluoride membranes. After blocking of nonspecific binding with TBS-T containing 5% nonfat milk for 1 h at room temperature, the membranes were immunoblotted with the primary antibodies at 4°C overnight. Antibodies to the following were used for western blot (dilution 1:1000): α-SMA (MAB1420, R&D Systems), PDGFR-β (ab69506, Abcam), and Desmin (ab32362, Abcam). The membranes were subsequently incubated with HRP-conjugated goat anti-rabbit or anti-mouse secondary antibodies for 2 h at room temperature. Protein bands were detected with ECL western blot reagent (Bioworld Technology).

### Frozen sectioning and immunofluorescence analysis of mouse tumors

The tumors were frozen in OCT embedding medium, sectioned (5 μm), fixed in 100% cold methanol for 20 min at room temperature, permeabilizated with 0.5% Triton X-100 for 30 min, and blocked with 5% BSA for 60 min^31^. The sections were incubated at 4°C overnight with anti-CD31 (ab7388, Abcam), anti-α-SMA (MAB1420, R&D Systems), anti-PDGFR-β (ab69506, Abcam), or anti-Desmin (ab32362, Abcam). The sections were then incubated with the corresponding secondary antibodies for 1 h at room temperature, mounted with DAPI Mounting Medium (Vector Laboratories), and analyzed with a Leica TCS SP5 confocal microscope.

### Statistical analysis

Statistical analyses were performed with unpaired Student’s t-test and two-way ANOVA. Data are reported as mean ± standard deviation. *P* values < 0.05 were considered statistically significant in all analyses: *P < 0.05, **P < 0.01, ***P < 0.001.

## Results

### TNFSF15 facilitates myeloid CD11b^+^ cell differentiation and proliferation

**Figure 1:**
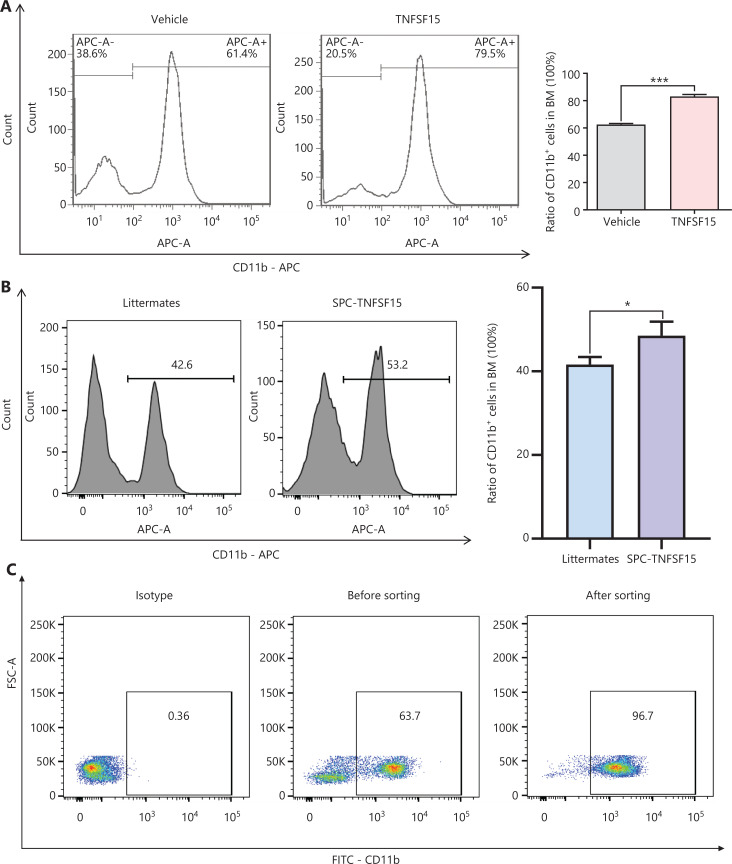

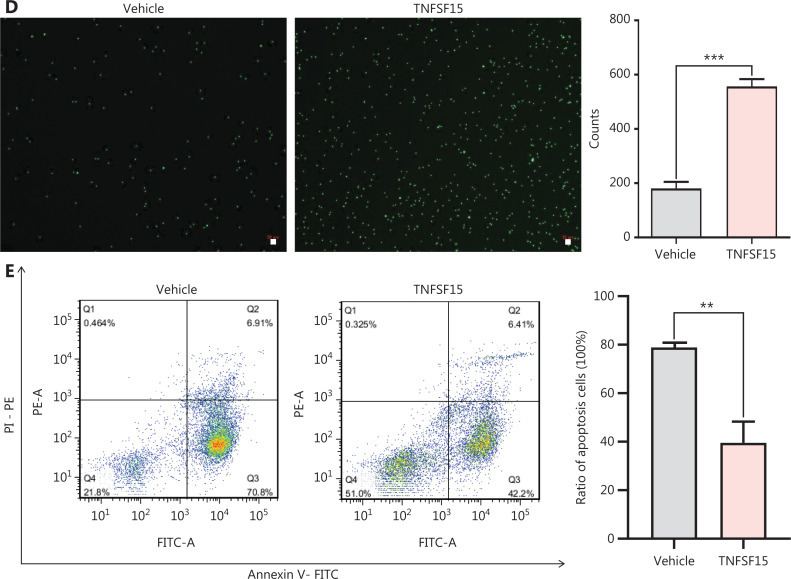
TNFSF15 facilitates myeloid CD11b^+^ cell differentiation and survival. (A) Representative images (left) and quantification (right) of flow cytometric analyses of CD11b^+^ cells isolated from the bone marrow of wild-type C57BL/6J mice with or without TNFSF15 treatment (5 mg/kg, *n* = 5). (B) Representative images (left) and quantification (right) of flow cytometric analyses of CD11b^+^ cells isolated from the bone marrow of SPC-TNFSF15 transgenic mice or their transgene-free littermates (*n* = 5). (C) Representative images of flow cytometric analyses of the purity of CD11b^+^ cells freshly isolated with magnetic beads from the bone marrow of wild-type C57BL/6J mice. (D) Calcein staining images (left) and quantification (right) of CD11b^+^ cells with or without TNFSF15 treatment (3 μg/mL) for 3 days. Scale bar: 50 μm. (E) Representative images (left) and quantification (right) of flow cytometry detection of apoptosis of CD11b^+^ cells with or without TNFSF15 treatment (3 μg/mL) for 3 days. Data were analyzed with unpaired Student’s t-test and are presented as means ± SD (*n* = 3, *P < 0.05, **P < 0.01, ***P < 0.001).

Prompted by our earlier findings that TNFSF15 modulates hematopoietic stem cell differentiation^28^, we treated wild-type C57BL/6J mice with recombinant TNFSF15 by intraperitoneal injection, isolated the bone marrow cells, and determined the composition of the myeloid cell population. TNFSF15 treatment led to a 1.3-fold increase in the population of CD11b^+^ cells in the bone marrow (**Figure 1A**). We also observed a similarly elevated CD11b^+^ cell population in the bone marrow in TNFSF15-overexpressing transgenic mice compared with transgene-negative littermates (**Figure 1B**). We then isolated CD11b^+^ cells from the bone marrow of wild-type C57BL/6J mice (**Figure 1C**) and cultured them in the presence or absence of TNFSF15. We found approximately 2 times more viable CD11b^+^ cells in TNFSF15-treated cultures than vehicle-treated cultures (**Figure 1D**). Additionally, the apoptosis rate of TNFSF15-treated CD11b^+^ cells was approximately 50% of that of vehicle-treated cells (**Figure 1E**). These data suggested that TNFSF15 facilitates production of CD11b^+^ cells in the bone marrow and exerts a protective effect on these cells in cultures.

### Treatment of tumor-bearing mice with TNFSF15 enhances CD11b^+^ cell accumulation in tumors

**Figure 2:**
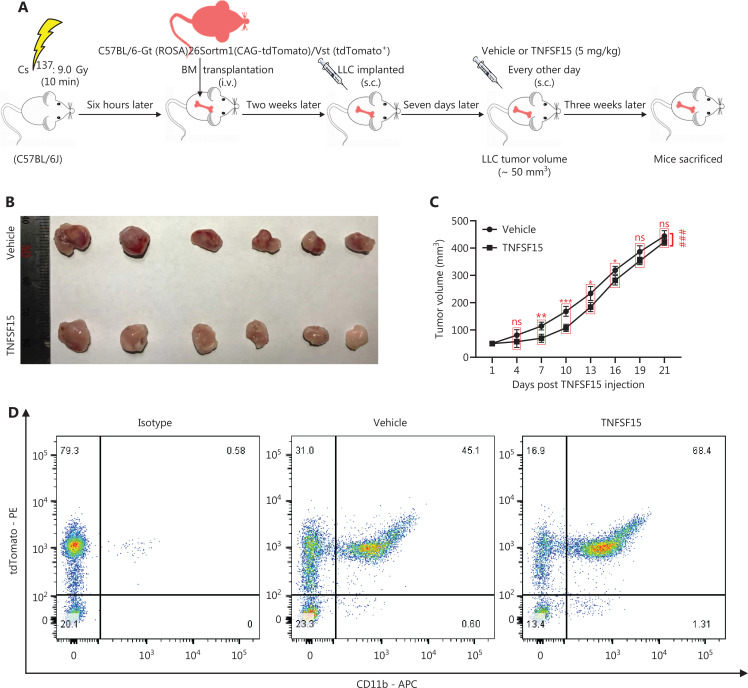

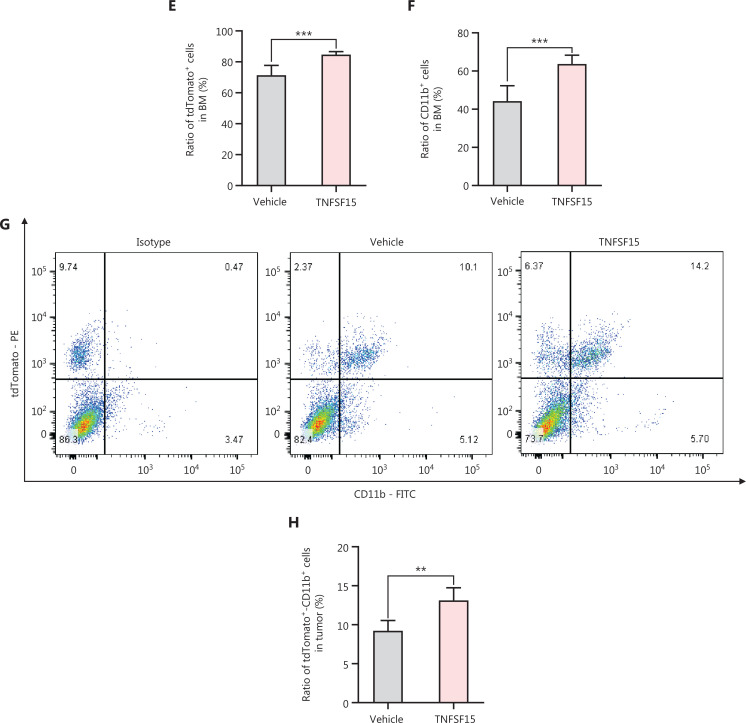
Treatment of tumor-bearing mice with TNFSF15 enhances CD11b^+^ cell accumulation in tumors. (A) Design and experimental model of the tdTomato bone marrow transplantation LLC tumor-bearing animal model. (B) Image of LLC tumors with or without TNFSF15 treatment (5 mg/kg) on day 21, *n* = 6. (C) Volume measurement of LLC tumors, *n* = 6. (D–F) Flow cytometric analysis and statistical analysis of tdTomato^+^ and CD11b^+^ cells in the bone marrow of the vehicle-treated group or TNFSF15-treated group. Flow cytometric analysis (G) and quantification (H) of tdTomato^+^-CD11b^+^ cells in vehicle- or TNFSF15-treated tumors. Data were analyzed with two-way ANOVA (*n* = 6, ^###^*P* < 0.001) or unpaired Student’s t-test, and are presented as mean ± SD (*n* = 6, *P < 0.05, **P < 0.01, ***P < 0.001).

We performed bone marrow transplantation to replace the bone marrow of the experimental animals with red fluorescent bone marrow from tdTomato-transgenic mice^34,35^, then implanted LLC tumors in the animals with tdTomato^+^ bone marrow, and treated them with recombinant TNFSF15 through intraperitoneal injection (5 mg/kg per injection) (**Figure 2A**). TNFSF15 treatment under these experimental conditions retarded tumor growth in the first 2 weeks of the treatment period (**Figure 2B, C**). Notably, TNFSF15 inhibition of tumor growth in animal models was often more effective in the first week^31,33^. Because the new blood vessels in the tumors were structurally inadequate^33^, we continued the experiments for about two weeks before collection of the specimens, to enable observation of more mature blood vessels with greater pericyte coverage. Analyses of the bone marrow cells (**Figure 2D**) indicated that the 70%–80% of the bone marrow cells had the fluorescent marker (**Figure 2E**), and the population of tdTomato^+^-CD11b^+^ double positive cells in the bone marrow in was approximately 52% greater in TNFSF15-treated animals than vehicle-treated animals(**Figure 2F**). Analyses of the population of the bone marrow-derived CD11b^+^ cells in the tumors revealed that the percentages of the tdTomato^+^-CD11b^+^ cells in the vehicle- and TNFSF15-treated groups were approximately 9% and 13%, respectively (**Figure 2G, H**). Thus, TNFSF15 treatment of the tumor-bearing animals enhanced the accumulation of CD11b^+^ cells in tumors.

### TNFSF15 promotes coverage of the tumor neovasculature by bone marrow-derived pericytes

**Figure 3:**
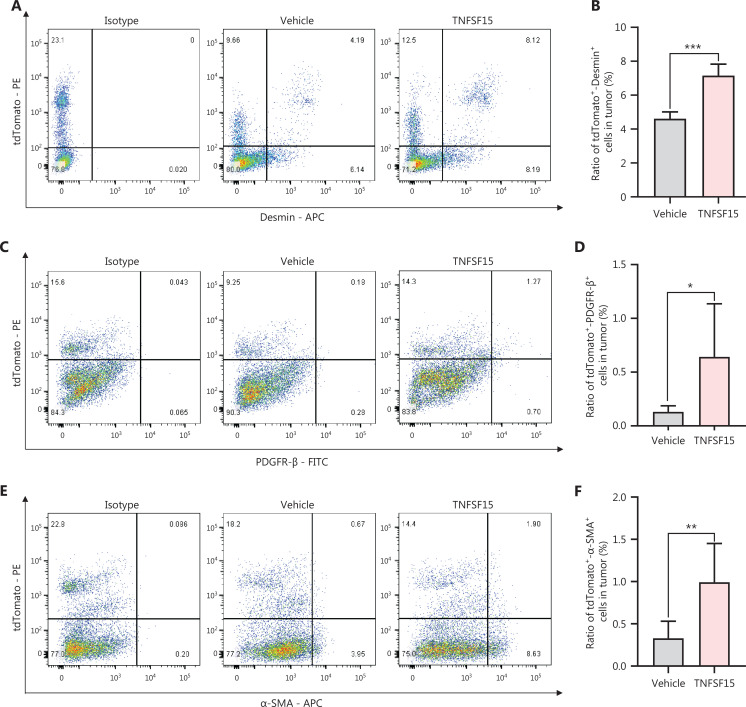

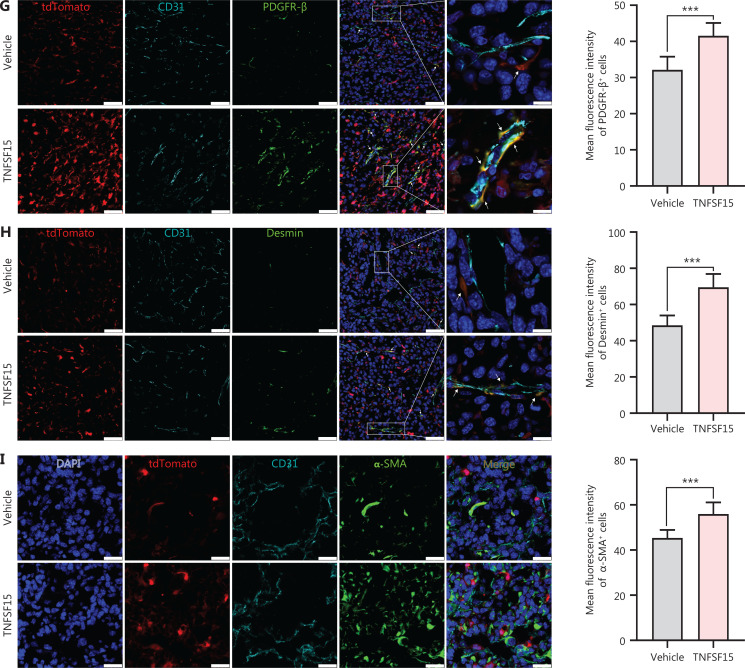
TNFSF15 promotes coverage of the tumor neovasculature by bone marrow-derived pericytes. Flow cytometric analysis and statistical analysis of tdTomato^+^-Desmin^+^ (A, B), tdTomato^+^-PDGFR-β^+^ (C, D), and tdTomato^+^-α-SMA^+^ cells (E, F) in vehicle- or TNFSF15-treated bone marrow transplantation LLC tumors. Data are presented as means ± SD (*n* = 6). *P < 0.05, **P < 0.01, ***P < 0.001. Confocal microscopic analysis of co-localization of vascular endothelial cells (CD31^+^), bone marrow-derived pericytes (tdTomato^+^-PDGFR-β^+^) (G), and (tdTomato^+^-Desmin^+^) (H) in tumor tissues. Scale bars: 50 μm and 7.5 μm (in zoom view). (I) Confocal microscopic analysis of co-localization of vascular endothelial cells (CD31^+^) and bone marrow-derived pericytes (tdTomato^+^-α-SMA^+^) in tumor tissues. Scale bar: 25 μm. Two visual fields for each tissue and 12 visual fields for each group were used in statistical analyses of the average immunofluorescence density. Data were analyzed with unpaired Student’s t-test and are presented as means ± SD (*n* = 6, *P < 0.05, **P < 0.01, ***P < 0.001).

Because bone marrow-derived CD11b^+^ cells infiltrating into tumors have been suggested to act as pericytes that stabilize tumor blood vessels^14^, we determined the percentage of tdTomato^+^ cells in the tumors that also displayed the smooth muscle cell/pericyte markers Desmin, PDGFR-β, or α-SMA. We found that tdTomato^+^-Desmin^+^ cells (**Figure 3A, B**), tdTomato^+^-PDGFR-β^+^ cells (**Figure 3C, D**), and tdTomato^+^-α-SMA^+^ cells (**Figure 3E, F**) in the tumors of the TNFSF15-treated group were 0.6-, 4-, and 2.3-fold higher, respectively, than those in the vehicle-treated group. Additionally, immunofluorescence staining of tumor sections for tdTomato^+^ vascular endothelial cells (CD31^+^) and smooth muscle cell/pericytes (PDGFR-β^+^, Desmin^+^, or α-SMA^+^) demonstrated markedly greater pericyte coverage of endothelial cells in the TNFSF15-treated group than the vehicle-treated group (**Figure 3G–I**). These findings indicated that TNFSF15 treatment of tumor-bearing mice enhanced the accumulation of bone marrow-derived pericytes and the coverage of the tumor neovasculature by these cells.

### TNFSF15 facilitates differentiation of bone marrow-derived CD11b^+^ cells into pericytes

**Figure 4:**
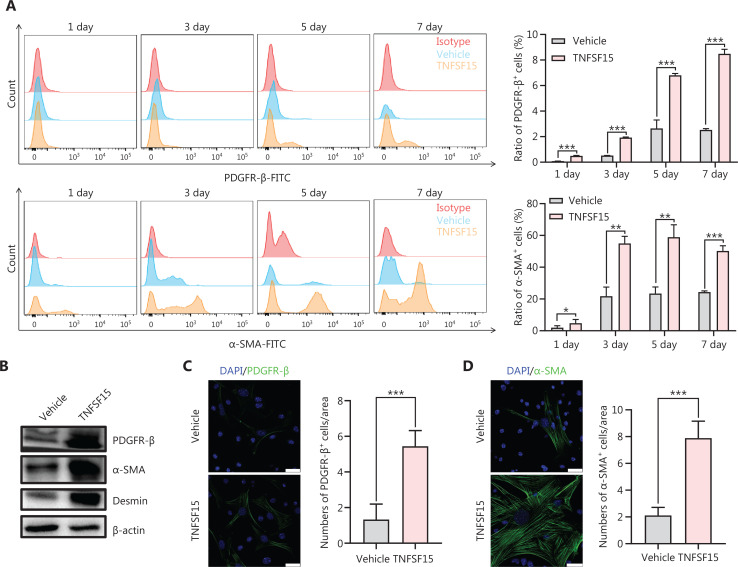

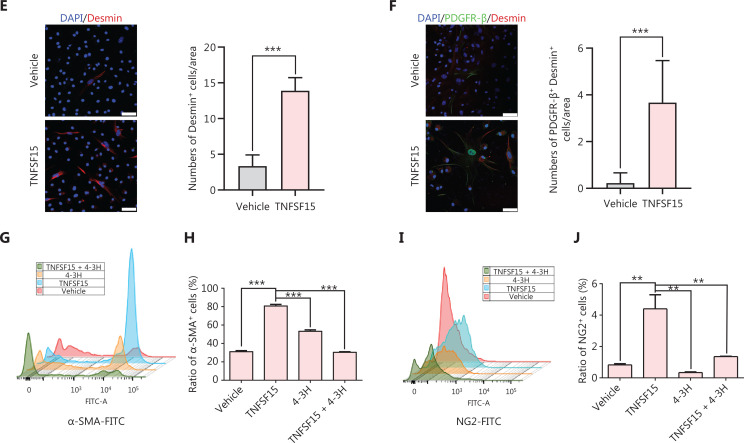
TNFSF15 facilitates bone marrow-derived CD11b^+^ cell differentiation into pericytes. (A) Flow cytometric analysis (left) and statistical analysis (right) of PDGFR-β^+^ cells and α-SMA^+^ cells differentiated from vehicle- or TNFSF15-treated myeloid CD11b^+^ cells for 1, 3, 5, or 7 days. (B) Western blot analysis of the levels of PDGFR-β, α-SMA, and Desmin in CD11b^+^ cells treated with vehicle or TNFSF15 for 3 days. (C–F) Typical images of immunofluorescence staining of CD11b^+^ cells treated with vehicle or TNFSF15 for 3 days. DAPI reagent was used to stain cell nuclei. Scale bar: 25 μm. Three visual fields of each tissue and 9 visual fields of each group were taken, and the number of cells was statistically analyzed according to the co-localization of nuclear DAPI and immunofluorescence. Data were analyzed with unpaired Student’s t-test and are presented as means ± SD (*n* = 3, *P < 0.05, **P < 0.01, ***P < 0.001). Flow cytometric analysis (G, I) and quantification (H, J) of α-SMA^+^ cells and NG2^+^ cells in CD11b^+^ cells treated with vehicle or TNFSF15 for 3 days. Data were analyzed with unpaired Student’s t-test and are presented as means ± SD (*n* = 3, *P < 0.05, **P < 0.01, ***P < 0.001).

We then freshly isolated CD11b^+^ cells from the bone marrow of wild-type C57BL/6J mice to determine whether recombinant TNFSF15 treatment might stimulate differentiation of these cells into pericytes. The percentages of PDGFR-β^+^ cells in TNFSF15-treated CD11b^+^ cell cultures, when analyzed on days 1, 3, 5, and 7, were approximately 4.0-, 3.7-, 2.6-, and 3.5-fold those in the vehicle-treated cultures, whereas the percentages of α-SMA^+^ cells in TNFSF15-treated cultures were approximately 3.1-, 2.6-, 2.5-, and 2.1-fold those in the vehicle-treated cultures (**Figure 4A**). Western blot analyses of the protein levels of PDGFR- β, α-SMA, and Desmin in CD11b^+^ cells cultured in the presence of TNFSF15 for 7 days confirmed the significantly greater expression of these the pericyte markers than observed in the vehicle-treated cells (**Figure 4B**). The up-regulation of pericyte markers in TNFSF15-treated CD11b^+^ cells was further confirmed by co-immunofluorescence staining of the cell cultures (**Figure 4C–F**). To corroborate these findings, we treated the CD11b^+^ cell cultures with a TNFSF15 neutralizing antibody, 4–3H, in the presence or absence of TNFSF15, and found that the percentage of α-SMA^+^ cells decreased by 1.7-fold when the activity of TNFSF15 was inhibited by the neutralizing antibody (**Figure 4G, H**). Furthermore, we repeated this experiment with another pericyte marker, NG2, and found that the percentage of NG2^+^ cells in CD11b^+^ cell cultures treated with TNFSF15 increased by 4.2-fold, whereas co treatment of the cells with 4–3H decreased the percentage of NG2^+^ cells to a level similar to that observed in vehicle-treated cell cultures (**Figure 4I, J**). These findings suggested that TNFSF15 facilitates bone marrow-derived CD11b^+^ cell differentiation into pericytes.

### TNFSF15 up-regulates the CD49e-fibronectin (FN) signaling pathway and down-regulates Wnt3a-VEGFR1 in CD11b^+^ cells

**Figure 5:**
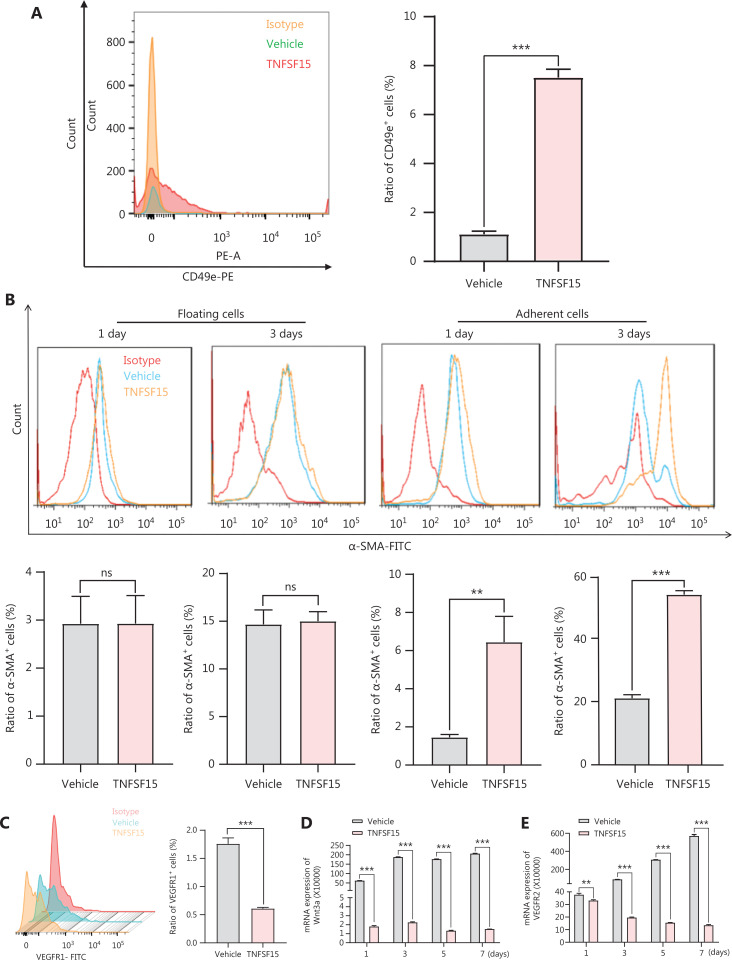
Up-regulation of the CD49e-fibronectin signaling pathway and down-regulation of Wnt3a-VEGFR1 in CD11b^+^ cells by TNFSF15. (A) Flow cytometry analysis (left) and statistical analysis (right) of the expression of CD49e in myeloid CD11b^+^ cells with or without TNFSF15 treatment (3 μg/mL) for 3 days. (B) Flow cytometry analysis of the expression of α-SMA in suspended and adherent myeloid CD11b^+^ cells with or without TNFSF15 treatment (3 μg/mL) for 1 or 3 days. (C) Flow cytometric analysis (left) and statistical analysis (right) of the proportion of VEGFR1 positive cells among myeloid CD11b^+^ cells with or without TNFSF15 treatment (3 μg/mL) for 3 days. RT-PCR analysis of the mRNA expression of Wnt3a (D) and VEGFR2 (E) in myeloid CD11b^+^ cells with or without TNFSF15 treatment (3 μg/mL) for 1, 3, 5, or 7 days. Each experiment was conducted 3 times independently, and the results are presented as means ± SD. ns, not significant (*P* > 0.05); Student’s t test was used to analyze the data (*n* = 3, **P < 0.01, ***P < 0.001).

Because integrin α5β1 and FN are critical adhesion signals in the recruitment and differentiation of mononuclear cell-derived vascular smooth muscle progenitor cells into smooth muscle cells^36,37^, we used flow cytometry to determine the expression of CD49e (integrin α5) in bone marrow-derived CD11b^+^ cells cultured on FN coating, to elucidate the molecular mechanism of TNFSF15-induced differentiation of pericytes from CD11b^+^ cells. The CD49e protein level in TNFSF15-treated cells was approximately 7 times that in vehicle-treated cells (**Figure 5A**). CD11b^+^ cells were subsequently divided into floating cells and adherent cells, and the percentages of α-SMA^+^ cells were determined in these 2 populations on day 1 and day 3 after cell seeding. No significant differences in the percentages of α-SMA^+^ were observed in the floating cells, regardless of TNFSF15 treatment (**Figure 5B**). Interestingly, however, the percentage of α-SMA^+^ cells in the adherent population of TNFSF15-treated CD11b^+^ cells was approximately 4.4- and 2.6-fold that in the vehicle-treated cells on day 1 and day 3, respectively (**Figure 5B**). These findings suggested that TNFSF15’s actions strengthen the CD49e-FN signaling pathway in CD11b^+^ cells and the ability of these cells to adhere.

We determined the effects of TNFSF15 treatment on CD11b^+^ cells regarding the expression of VEGFR1, VEGFR2, and the Wnt signaling pathway protein Wnt3a. Because VEGF-induced activation of VEGFR1 is critical in promoting neovascularization, we determined whether TNFSF15 might inhibit VEGFR1 gene expression in this model, as in the models we analyzed previously^38^. Activation of the Wnt3a-VEGFR1 signaling pathway has been shown to inhibit the adipogenic differentiation of pericytes in blood vessels^39^, and activation of VEGFR2 has been found to decrease pericyte coverage of blood vessels^40^. We found that VEGFR1 protein levels were nearly two-thirds lower in TNFSF15-treated cells than. vehicle-treated cells (**Figure 5C**). We then determined the mRNA levels of Wnt3a and VEGFR2 in CD11b^+^ cells with or without TNFSF15 treatment for various time intervals. The mRNA levels of Wnt3a (**Figure 5D**), and VEGFR2 (**Figure 5E**) were much greater in vehicle-treated CD11b^+^ cells than in TNFSF15-treated cells. These findings were consistent with TNFSF15 treatment inhibiting Wnt3a-VEGFR1 signals and VEGFR2 activation, and consequently enhancing the association of pericytes with vascular endothelial cells.

## Discussion

**Figure 6:**
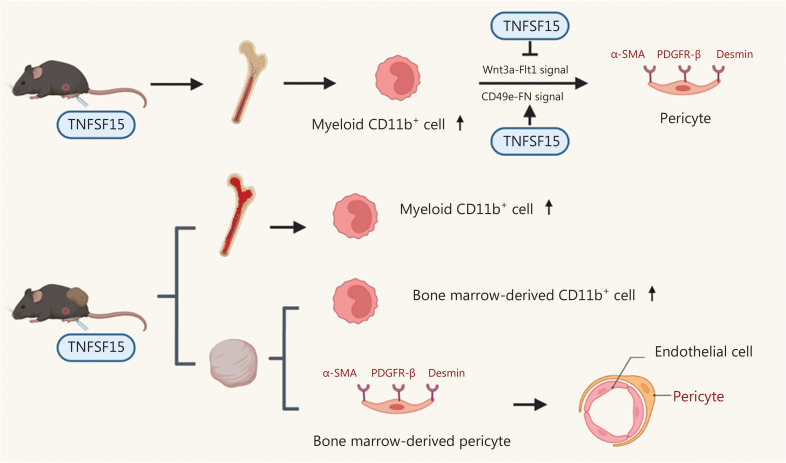
Schematic of the mechanisms underlying TNFSF15 modulation of CD11b^+^ cell differentiation to vascular pericytes. TNFSF15 stimulates CD11b^+^ cell production in the bone marrow and facilitates bone marrow-derived CD11b^+^ cell differentiation into pericytes in cell cultures. Down-regulation of the Wnt3a-VEGFR1 signaling pathway and up-regulation of the CD49e-FN signaling pathway by TNFSF15 are involved. Treatment of tumor-bearing mice with recombinant TNFSF15 enhances the association of bone marrow-derived pericytes with endothelial cells in tumor vessels.

Our findings demonstrated that TNFSF15 promotes the differentiation of bone marrow-derived myeloid cells into CD11b^+^ cells and vascular pericytes (**Figure 6**). This process also occurs *in vivo*, because treatment of the mouse LLC tumor model with TNFSF15 enhanced the CD11b^+^ cell population percentage in the bone marrow, and significantly increased the percentage of tumor-infiltrated pericytes and their association with vascular endothelial cells. TNFSF15 had anti-apoptosis effects on CD11b^+^ cells isolated from the bone marrow and cultured under our experimental conditions. Mechanistically, TNFSF15 treatment of CD11b^+^ cells up-regulated CD49e-FN signaling pathways and down-regulated the Wnt3a-VEGFR1 signaling pathways, in agreement with enhanced ability of the resultant pericytes to adhere and associate with vascular endothelial cells (**Figure 6**).

We previously demonstrated that TNFSF15 inhibits vasculogenesis by regulating relative levels of membrane-bound and soluble isoforms of VEGFR1^32^, and inhibits VEGF-stimulated vascular hyperpermeability by inducing VEGFR2 dephosphorylation^25^. In this study, we demonstrated that gene expression of both VEGFR1 and VEGFR2 in CD11b^+^ cells was down-regulated by TNFSF15. In contrast, the pericyte markers a-SMA^41^, PDGFR-β^42^, and Desmin^43^ in CD11b^+^ cells were up-regulated by TNFSF15. These findings are consistent with TNFSF15 directing the differentiation of CD11b^+^ cells toward pericytes, thus promoting neovasculature stabilization.

Substantial experimental evidence supports that vascular smooth muscle cells can be used to stabilize the neovasculature. Enhanced pericyte coverage of the tumor neovasculature decreases vascular permeability, thus lowering interstitial fluid pressure in tumor tissues and improving perfusion^44–47^; consequently, diffusion of blood-borne molecules into the tumor interstitium is enhanced^48^, and the delivery of systemically administered therapeutic agents^49,50^ and the infiltration of immune cells^51–55^ are accelerated. Our findings that TNFSF15 facilitates the differentiation of bone marrow-derived CD11b^+^ cells to vascular pericytes not only yield new insights into the molecular mechanisms underlying neovascularization but also provide a potentially feasible approach to modifying the tumor microenvironment to increase the efficacy of cancer therapies.

The process of tumor neovascularization has been likened to a wound failing to heal, with continuous cycling of vascular vessel growth, exudation of tissue fluid, and invasion of mesenchymal cells^56^. Consequently, the tumor vessels have irregular diameters, frequent intussusception, and irregular branching patterns. These vessels have incomplete basement membranes and discontinuous pericyte coatings^57^. The aberrant structure and function of the tumor vascular system not only stimulate ongoing angiogenic processes, but also give rise to continuous vascular leakage, elevated interstitial hydraulic pressure, and persistent inflammatory conditions. Tumor blood vessel normalization or stabilization would lead to lower interstitial hydraulic pressure in tumors, thereby improving drug delivery to tumors^58^. Moreover, cancer cell growth markedly slows after tumor neovasculature stabilization by enhanced pericyte coverage^9^, probably because the tumor growth rate is limited by the vascular growth rate. Moreover, a stabilized tumor vasculature might plausibly hinder the escape of cancer cells from tumors.

In summary, we demonstrated that TNFSF15 promotes differentiation of freshly isolated myeloid cells into CD11b^+^ cells and further into pericytes. Additionally, treatment of a mouse tumor model containing red fluorescent bone marrow increased CD11b^+^ myeloid cells and vascular pericytes in the tumors. Mechanically, TNFSF15 protects CD11b^+^ cells against apoptosis, and enhances the adhesion ability of these cells by down-regulating Wnt3a-VEGFR1 and up-regulating CD49e-FN signaling pathways. These findings support that TNFSF15 facilitates the production of CD11b^+^ cells in the bone marrow and promotes the differentiation of these cells into pericytes, which may stabilize the tumor neovasculature.

## Data Availability

The data that support the findings of this study are available from the corresponding author.
