# Supplementary material for: TNFSF15 facilitates the differentiation of CD11b+ myeloid cells into vascular pericytes in tumors
Source: Cancer Biol Med. 2023 Nov 2;20(11):869–84. doi: 10.20892/j.issn.2095-3941.2023.0245 (PMC10690882; doi:10.20892/j.issn.2095-3941.2023.0245)
Supplement: Supplementary file 1 [file cbm-20-869-s001.pdf]

Supplementary material

Table S1 Names and sequences of primers used for RT-PCR

Name	Sense (5′-3′)	Antisense (5′-3′)
Wnt3	GATCCCAGTCGCGCGATC	GAGCCAGGTTTAGGGAGCTG
VEGFR1	CACTGACATACCCAAACTTGTGC	GTCCCATGTTATTCTTTGCCCAT
VEGFR2	AGAAGTGGCAACGGCACCT	CAAAGCATTGCCCATTCGAT
GAPDH	AGGTCGGTGTGAACGGATTTG	TGTAGACCATGTAGTTGAGGTCA
β-Actin	GAAGTGTGACGTTGACATCCGTA	CTCAGGAGGAGCAATGATCTTGA
